# Prognostic Value of Pretreated Blood Inflammatory Markers in Patients with Bone Sarcoma: A Meta-Analysis

**DOI:** 10.1155/2021/8839512

**Published:** 2021-04-08

**Authors:** Min Jiang, Shaowei Ma, Zhongyan Hua, Zhiying Zhao, Song Gao

**Affiliations:** ^1^Department of Oncology, Shengjing Hospital of China Medical University, Shenyang 110004, China; ^2^Department of Cardiology, Shengjing Hospital of China Medical University, Shenyang 110004, China; ^3^Medical Research Center, Liaoning Key Laboratory of Research and Application of Animal Models for Environmental and Metabolic Diseases, Shengjing Hospital of China Medical University, Shenyang 110004, China; ^4^Department of Clinical Epidemiology, Shengjing Hospital of China Medical University, Shenyang 110004, China

## Abstract

**Method:**

We conducted a detailed literature search in Medline and Embase databases and collected relevant publications written in English before April 2020. Overall survival (OS) and disease-free survival (DFS) were the primary and secondary outcomes, respectively. Basic features of patients, hazard ratios (HRs), and 95% confidence intervals (CI) were retrieved to assess the correlation between pretreated blood inflammatory markers and patients with bone sarcoma. This meta-analysis used Stata 12.0.

**Results:**

A total of 10 studies containing 1845 cases were included for analysis. Nine of them evaluated the neutrophil lymphocyte ratio (NLR), 7 the platelet lymphocyte ratio (PLR), and 4 the lymphocyte monocyte ratio (LMR). Pooled results revealed that higher pretreatment NLR was associated with poorer OS (HR = 1.76, 95% CI: 1.29–2.41, and *P* < 0.001) and DFS (HR = 1.77, 95% CI: 1.09–2.88, and *P* = 0.021). In contrast, a lower LMR was related to worse OS (HR = 0.73, 95% CI: 0.57–0.92, and *P* = 0.009), but not DFS (HR = 0.68, 95% CI: 0.41–1.11, and *P* > 0.05). Combined results did not show a significant predictive effect of PLR on the clinical outcomes of patients with bone sarcoma (OS : HR = 1.32, 95% CI: 0.99–1.75, and *P* > 0.05; DFS: HR = 1.12, 95% CI: 0.87–1.44, *P* > 0.05).

**Conclusion:**

NLR and LMR might be promising predictive biomarkers for patients with bone sarcoma and could be used to stratify patients and provide personalized therapeutic strategies.

## 1. Introduction

Sarcoma refers to malignancies originating from mesenchymal tissues and can be classified as bone or soft tissue sarcoma [1]. Bone sarcoma is a rare group of tumors, mainly consisted of osteosarcoma, Ewing sarcoma, and chondrosarcoma [2]. Osteosarcoma and Ewing sarcoma share similar clinical epidemiological characteristics, primarily affecting children and adolescents [3, 4]. The incidence rate of chondrosarcoma has been shown to exhibit a gradual increase with age [4]. Although the incidence of bone sarcoma among the whole population is known to be relatively low (less than 0.2% of all new cancers), it accounts for 6% of all childhood cancers, presenting a high rate of fatality and overall disease burden [4]. Treatment of bone sarcomas in clinical practice has been challenging. Despite combined modality treatment protocols consisting of surgery, chemotherapy, and radiation, the outcomes of patients have not significantly improved for decades. Relapse rates have been reported to remain high at about 35% [5], with the 5-year survival rate for cases with metastasis being as low as 10-30% [5, 6]. Prognosis prejudgment is essential for clinical decision-making. However, effective prognostic biomarkers for patients with bone sarcoma are still lacking. Therefore, identifying novel parameters to effectively predict prognosis and to help clinicians with the treatment option is of grave importance.

Accumulating evidence has revealed that cancer-associated systematic inflammation might play crucial roles in the genesis and progression of tumors [7–10]. The inflammatory response is known to be reflected by many blood biomarkers, including neutrophil lymphocyte ratio (NLR), platelet lymphocyte ratio (PLR), and lymphocyte monocyte ratio (LMR). These biomarkers can be easily calculated with a routine blood test, which is both convenient and economic. Recently, several retrospective studies have reported the prognostic value of these inflammatory biomarkers for patients with bone sarcoma, but no consensus has been reached so far. Therefore, this study is aimed at investigating the role of pretreatment blood inflammatory biomarkers on the prognosis of patients with bone sarcoma.

## 2. Materials and Methods

### 2.1. Search Strategy

The search was based on the Preferred Reporting Items for Systematic Reviews and Meta-Analyses (PRISMA) guidelines. We conducted a systematic electronic search of the Medline and Embase databases up to April 2020. Different combinations of keywords used for preliminary search were as follows: “neutrophil” or “neutrophils,” “lymphocyte” or “lymphocytes,” “platelet” or “platelets,” “monocyte” or “monocytes,” “osteosarcoma” or “Ewing sarcoma” or “chondrosarcoma” or “bone sarcoma,” and “prognostic” or “prognosis” or “outcome” or “survival.” As this was a meta-analytic study using data only from published studies, ethical approval was waived.

### 2.2. Inclusion and Exclusion Criteria

Inclusion criteria are listed as follows: (1) pathological diagnosis of bone sarcoma; (2) studies assessing the association of NLR or PLR or LMR or their combinations with overall survival (OS), cancer-specific survival (CSS), or recurrence-free survival (RFS); (3) accessible hazard ratio (HR) and related 95% confidence interval (CI); (4) studies published in English; and (5) human studies. Exclusion criteria were as follows: (1) comments, reviews, case reports, conference abstracts, letters, and editorials; (2) studies containing subjects with soft tissue sarcomas; (3) studies with sample size smaller than 30; (4) overlapping or duplicate studies; (5) irrelevant studies; and (6) studies not in English.

### 2.3. Data Extraction and Quality Assessment

Two investigators (ZH and SM) reviewed the titles and abstracts of the articles identified in the initial search. Any discrepancy would be discussed, and a third reviewer (SG) would join in to reach consensus. The information extracted was as follows: name of first author, publication year, country, number of patients, presence or absence of metastatic patients, cutoff value, biomarker, survival outcomes, histology types, and analysis method. The HR and 95% CI values were preferentially collected from multivariate analysis; if no relevant data were offered, univariate analysis was considered as the alternative. Two investigators assessed the quality of preliminary screening articles according to the Newcastle-Ottawa scale (NOS). Studies with NOS scores ≥ 6 were considered high-quality and included in this meta-analysis [11].

### 2.4. Statistical Analysis

Considering their similarity, we combined event-free (EFS), progression-free (PFS), and disease-free (DFS) survival as DFS. Hazard ratios and 95% CI were applied to estimate the correlation of blood inflammatory markers and survival. The heterogeneity among studies was assessed by means of *Q*-test and *I*^2^ of the chi-square test. If significant heterogeneity (*P* < 0.05 and *I*^2^ > 50%) was observed, the random effect model was used; otherwise, the fixed effect model was employed. To identify the sources of heterogeneity, we performed subgroup analysis by tumor stage, analysis method, histological type, and ethnicity. Publication bias was conducted by means of the Begg test (funnel plots). The Stata software (Stata corporation, version 12.0, College Station, TX, USA) was used for the analysis of data, and statistical significance was considered for *P* values <0.05.

## 3. Results

### 3.1. Search Results and Characteristics of the Included Studies

**Figure 1:**
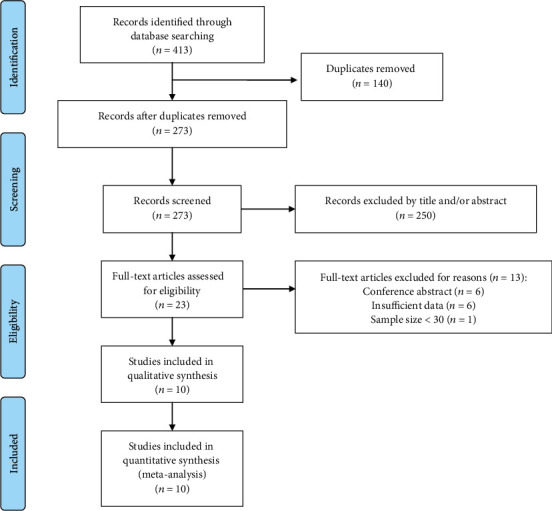
PRISMA flowchart of record search and selection.

A total of 413 articles were retrieved by our initial search of the Medline and Embase databases using our search strategy. After removing duplicates, 273 articles were left to be screened. Then, 250 articles were excluded by initial review, and only 23 were further assessed for eligibility. Among them, 6 reports were excluded because they were conference abstracts, whereas another 6 did not provide sufficient data to calculate the HR of patients with bone sarcoma and 1 article with only 23 samples. At last, 10 studies were included for quantitative synthesis [12–21]. The flow chart of the literature selection process is shown in Figure 1. As the study by Vasquez et al. [16] included both osteosarcoma and Ewing sarcoma cohorts, separately reporting the HR and 95% values, we marked the study of the Ewing sarcoma cohort as Vasquez et al.-EW. Similarly, the group of Li et al. reported the effect of inflammatory markers on the prognosis of patients with osteosarcoma and Ewing sarcoma in 2 articles of the same year [15, 17]; accordingly, we marked the Ewing sarcoma cohort as Li et al.-EW.

The publication time of 10 studies ranged from 2015 to 2020. Eight studies were from China, whereas the other 2 were from Peru and Denmark, respectively. The cutoff values of inflammatory markers were as follows: 2 to 5.3 for NLR, 118 to 200 for PLR, and 3.43 to 4.73 for LMR. The NOS score values ranged from 6 to 8. Detailed characteristics and quality assessment of eligible studies are shown in Table 1.

### 3.2. Meta-Analysis Results

#### 3.2.1. Correlation between Neutrophil Lymphocyte Ratio and Survival Outcomes in Bone Sarcomas

**Figure 2:**
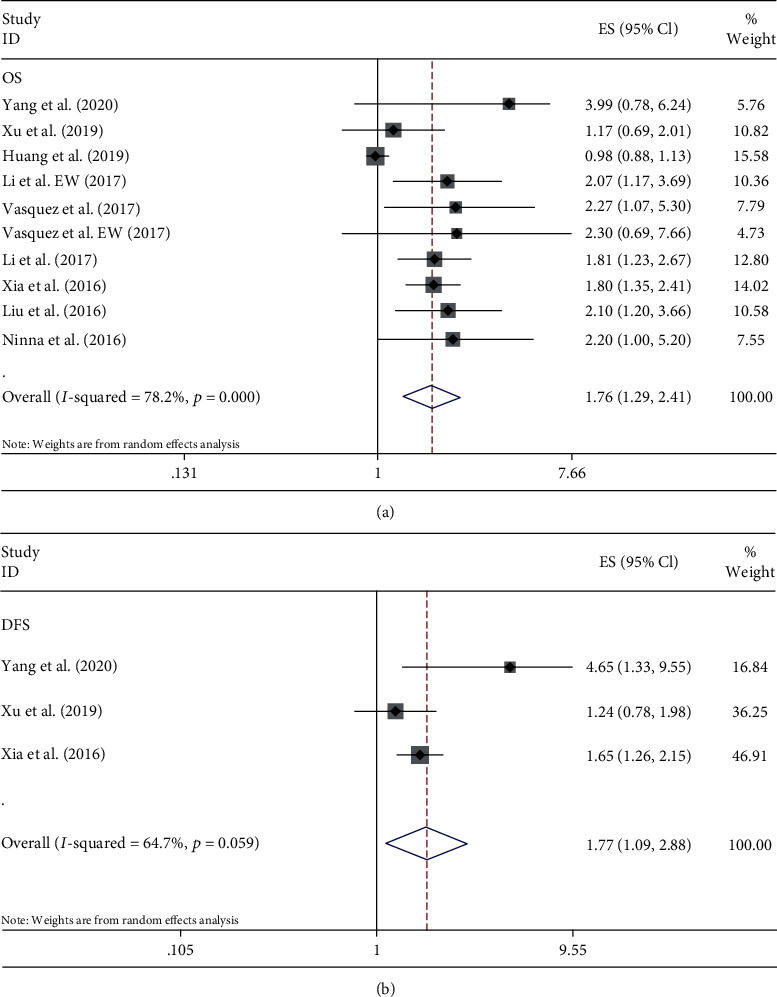
Forest plots of the prognostic effect of NLR for (a) OS and (b) DFS.

A total of 1518 patients with bone sarcomas were included in 9 studies investigating the prognostic value of NLR. All studies reported the relationship between NLR and OS. Among them, 3 studies provided the DFS, as well. As these studies were characterized by significant heterogeneity among them (OS: *P* < 0.05 and *I*^2^ = 78.2%; DFS: *P* = 0.059 and *I*^2^ = 64.7%), the random effect model was used to analyze both OS and DFS. These results revealed that the elevated NLR was significantly correlated with poorer OS (HR = 1.76, 95% CI: 1.29–2.41, and *P* < 0.001) (Figure 2(a)) and poorer DFS (HR = 1.77, 95% CI: 1.09–2.88, and *P* = 0.021) (Figure 2(b)).

Subgroup analysis illustrated that NLR was associated with poor OS in most conditions, except for chondrosarcoma and studies not including metastatic cases. This predictive connection was not observed to be affected by either ethnicity or the method of analysis. Details are shown in Table 2.

#### 3.2.2. Correlation between Platelet Lymphocyte Ratio and Survival Outcomes in Bone Sarcomas

**Figure 3:**
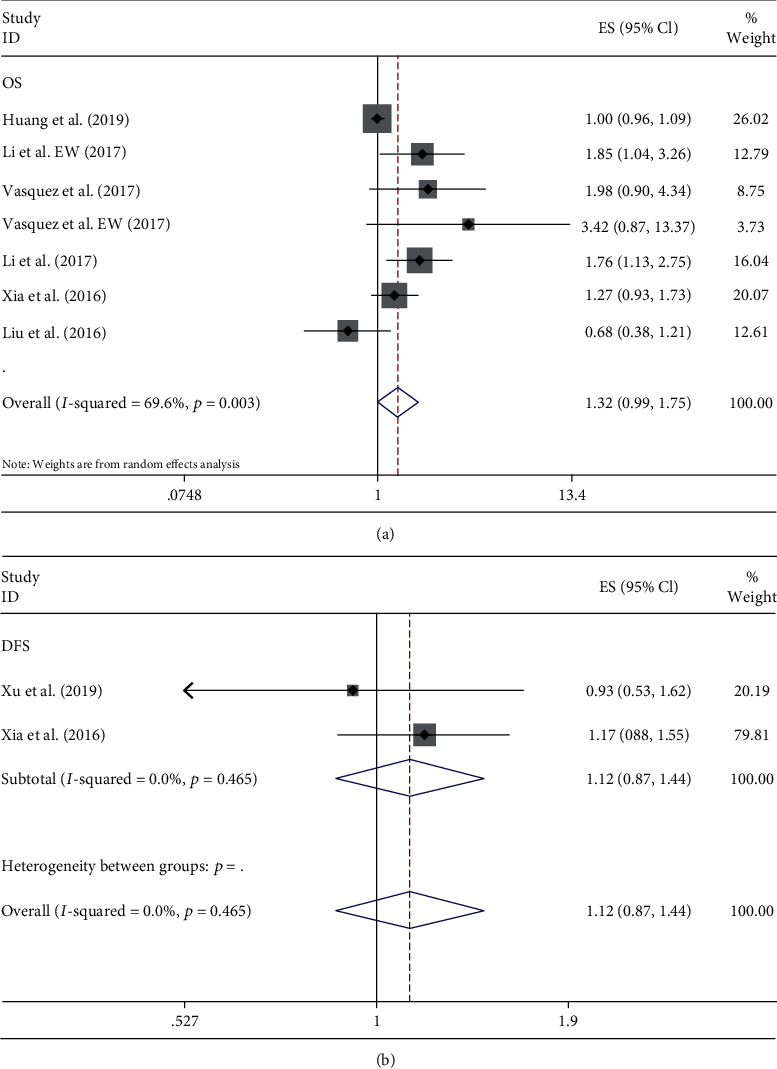
Forest plots of the prognostic effect of PLR for (a) OS and (b) DFS.

Seven studies of 1213 patients with bone sarcoma reported the prognostic role of PLR for OS, with 2 of them reporting DFS. We noted a significant heterogeneity among studies for OS (*P* < 0.05 and *I*^2^ = 69.6%), but no significant heterogeneity for DFS (*P* > 0.05 and *I*^2^ = 0). The random and fixed effect models were used for OS and DFS analyses, respectively. Results showed that the increase of PLR was not significantly related with either lower OS (HR = 1.32, 95% CI: 0.99–1.75, and *P* > 0.05) (Figure 3(a)) or lower DFS (HR = 1.12, 95% CI: 0.87–1.44, and *P* > 0.05) (Figure 3(b)).

Although pooled results showed no correlation between PLR and OS in patients with bone sarcoma, subgroup analysis revealed that elevated PLR was correlated with poor OS in patients with Ewing sarcoma, non-Asian patients, and studies analyzed by the univariate method. Details are shown in Table 3.

#### 3.2.3. Correlation between Lymphocyte Monocyte Ratio and Survival Outcomes in Bone Sarcomas

**Figure 4:**
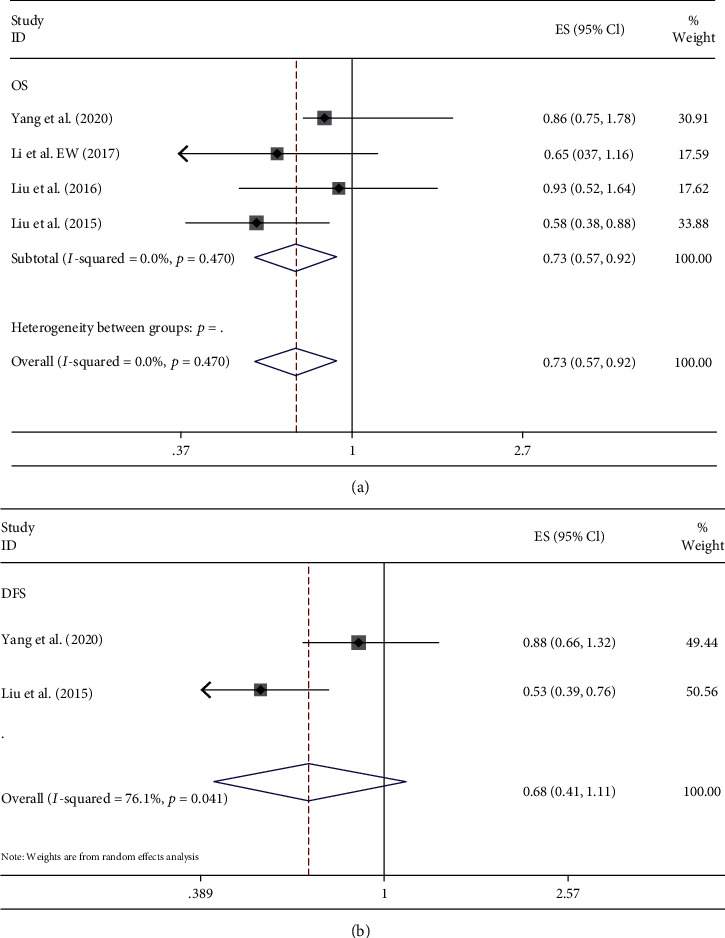
Forest plots of the prognostic effect of LMR for (a) OS and (b) DFS.

Four studies of 744 patients with bone sarcoma studied the prognostic effect of LMR on OS, with 2 of them reporting DFS. The fixed effect model showed that the decrease of LMR was associated with poor OS (HR = 0.73, 95% CI: 0.57–0.92, and *P* = 0.009), not revealing significant heterogeneity (*P* > 0.05 and *I*^2^ = 0) (Figure 4(a)). In contrast, we observed significant heterogeneity between studies for DFS (*P* < 0.05 and *I*^2^ = 76.1%). Analysis with the random effect model showed that the level of LMR was not related with DFS (HR = 0.68, 95% CI: 0.41–1.11, and *P* > 0.05) of patients with bone sarcoma (Figure 4(b)).

### 3.3. Publication Bias

**Figure 5:**
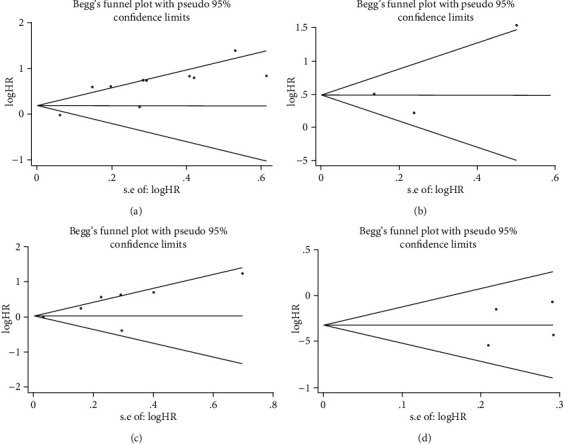
Analysis of publication bias for the relationship between inflammatory markers and prognosis in bone sarcoma. (a) Begg's funnel plot for NLR and OS. (b) Begg's funnel plot for NLR and DFS. (c) Begg's funnel plot for PLR and OS. (d) Begg's funnel plot for LMR and OS.

The funnel plots presented in Figure 5 revealed a slight publication bias of NLR for OS (Egger's test: *P* = 0.002; Begg's test: *P* = 0.721), but no publication bias of NLR for DFS (Egger's test: *P* = 0.615; Begg's test: *P* = 1.0). We subsequently calculated the new HR values using trim and fill methods (HR: 1.647; 95% CI: 1.232–2.201; *P* = 0.001; random effects), which further indicated the prognostic value of NLR for OS. There was no evidence of publication bias in the meta-analysis of the PLR value and OS. The Begg's and Egger's *P* values for PLR were 0.584 and 0.067, respectively. No potential publication bias was observed in the prognostic value of LMR and OS, as well (Egger's test: *P* = 0.657; Begg's test: *P* = 0.734).

## 4. Discussion

The Enneking and TNM staging systems have been serving as the foundation for predicting the prognosis of patients and determining proper treatment in bone sarcomas for a long time. However, these staging systems are known to only focus on the features of the tumor itself, such as its biological behavior, location, and size, without treating the malignant tumor as a systematic disease. As patients of the same tumor stage might vary in their clinical outcomes, current staging systems are not enough to precisely predict the prognosis of patients with bone sarcoma. Exploring laboratory parameters as potential prognostic markers might be a good strategy in complementing the existing staging system for improved stratification of patients.

The systemic inflammatory response has been suggested to have a crucial role in the development and progression of malignancies. Despite advanced progress in understanding the association between inflammatory biomarkers, such as NLR, PLR, LMR, and the prognosis of various cancers [22–25], the impact of inflammatory markers on the clinical prognosis of patients with bone sarcoma remains obscure. To our knowledge, this is the first attempt to appraise the correlation between NLR, PLR, LMR, and prognosis of patients with bone sarcoma. In our study, 3 major histological subtypes were included: osteosarcoma, Ewing sarcoma, and chondrosarcoma. Pooled results indicated that both NLR and LMR, but not PLR, were associated with the survival of patients with bone sarcoma. Our results showed that elevated pretreated NLR had an unfavorable influence on both OS and DFS in bone sarcomas. On the other hand, the decreased level of LMR was shown to be associated with poorer OS without any exhibited heterogeneity. The prognostic value of NLR for OS was not observed to be attenuated by subgroup analysis of NLR for OS based on ethnicity, histology types, and analytical method. Taken all these into consideration, our results suggested that NLR and LMR might serve as helpful prognostic markers in bone sarcomas.

The inflammatory response could cause neutrophilia, thrombocytosis, and lymphopenia [26]. Neutrophils have been considered as the major source of the vascular endothelial growth factor (VEGF), which is known to be a critical factor in tumor angiogenesis [27]. Besides VEGF, neutrophils are known to secrete other tumor-promoting factors, including hepatocyte growth factor [28], interleukin-8 (IL-8), IL-6 [29], and tumor necrosis factor [29], creating a favorable microenvironment for tumor survival. These neutrophil-induced inflammatory cytokines have been also demonstrated to help tumor cells subvert immune surveillance [30]. Elevated levels of platelets have been reported to not only accelerate angiogenesis of tumor and prevent cytolysis [31, 32], but also act as a chemoattractant in promoting the migration of cancer cells [33]. Tumor-associated macrophages, mainly originating from monocytes, have also been shown to induce angiogenesis, metastasis, and immune-suppression of tumor [34, 35], whereas lymphocyte-dependent cellular immune response is known to be of great importance in the immunological destruction of cancer cells [36]. Elevated lymphocyte infiltration in the tumor site has been suggested to be associated with favorable outcomes [37, 38]. In contrast, lymphopenia implied the impairment of the host immune response to tumor and was reported to be correlated with the severity of diseases [39, 40]. Because of all this background of the inflammatory response, inflammation-based prognostic indicators have emerged in the clinical management of patients with cancer.

Due to the low incidence, few studies are specifically aimed at exploring the prognostic value of inflammatory markers in bone sarcomas. Previous studies and meta-analyses have reported the prognostic role of different inflammatory markers in soft tissue sarcomas (STSs) [41, 42]. Elevated NLR and PLR and reduced LMR were demonstrated to be correlated with poor clinical outcomes in patients with STS [43, 44]. However, most studies have analyzed STSs together with osteoblastic tumors. Considering their difference, we separated osteoblastic tumors from STSs in this meta-analysis and got consistent results with previous studies. Although no statistical significance was observed, PLR still tended to predict poor OS with a *P* value of 0.059.

Several studies have indicated that the C-reactive protein (CRP) and the Glasgow prognostic score were also important inflammatory prognostic indicators [15, 45]. However, CRP was not a routine examination as part of the pretreatment assessment of patients with bone sarcoma in many hospitals. In comparison, NLR, PLR, and LMR were easy to be obtained just by performing a routine blood test. In recent years, noncoding RNAs have also been reported to be associated with the clinical prognosis of patients with bone sarcoma [46, 47].

However, the higher cost for the detection of noncoding RNAs has limited its general application in clinical practice. The inflammatory markers in our study possess the advantage of low cost and easy accessibility, which could be suitable for routine monitoring in predicting the clinical outcome in patients with bone sarcomas.

Despite all the advantages, the values of NLR, PLR, and LMR would be altered by certain diseases (such as infections, cardiac events, atherosclerosis, and abnormal thyroid function) and drugs like nonsteroidal anti-inflammatory drugs (NSAIDS). Although all the enrolled studies have claimed to exclude patients with diseases/drugs mentioned above, it was unavoidable to include patients with other underlying diseases that may also cause changes in these inflammatory biomarkers. Therefore, besides independent prognostic effects of these inflammatory biomarkers, novel index combining specific inflammatory biomarkers for different cancers (e.g., NLR and LMR for bone sarcomas based on our analysis) to assess their synergistic effects should be further studied as well.

This study had also some limitations that should be clarified. First, the number of studies included in this meta-analysis was not large, and only literature published in English was selected. Second, all included studies were retrospective, observational ones without the data of prospective cohorts. This might have resulted in bias in data analysis. Third, considering that outcomes might vary greatly depending on histological types, we performed subgroup analysis in osteosarcoma, Ewing sarcoma, and chondrosarcoma. Our results revealed the prognostic value of NLR in osteosarcoma and Ewing sarcoma, but not in chondrosarcoma. Therefore, studies on specific histological types are still needed to verify our results. Fourth, among all included studies, only 3 provided univariate data, which might have caused potential overestimation of the prognostic role of NLR and LMR. Besides, in the ten studies enrolled for quantitative synthesis, eight of them were from China and it might affect the generalisation of this work. Finally, as heterogeneity was observed among the included studies, we tried to identify the source by performing subgroup analysis. However, the prognostic value of NLR was demonstrated to not be affected in most subgroups, reinforcing the predictive effect of NLR and LMR in the clinical outcomes of patients with bone sarcomas.

## 5. Conclusions

Our meta-analysis demonstrated that higher pretreatment NLR and lower pretreatment LMR were strongly associated with poor prognosis in patients with bone sarcomas. Due to the low cost, high availability, and reproducibility, these inflammatory markers could be used to stratify high-risk patients with bone sarcomas and to provide suitable management strategies. However, to determine the optimal cutoff values of NLR and LMR in the prognosis of bone sarcoma still requires further prospective studies.

## Figures and Tables

**Table 1:** Basic characteristic of included study.

Author	Year	Country	Sample size	Stage	Cutoff value	Markers	Outcome	Histology type	Analysis method	NOS score
Yang et al.	2020	China	133	Mixed	2.96; 4.44	NLR, LMR	OS, PFS	Osteosarcoma	MA	8
Xu et al.	2019	China	150	Mixed	2.7; 200	NLR, PLR	OS, DFS	Chondrosarcoma	MA	8
Huang et al.	2019	China	126	Mixed	2.1; 163.2	NLR, PLR	OS	Osteosarcoma	MA	7
Li et al.	2017	China	122	Mixed	2.38; 131 4.41	NLR, PLR LMR	OS	Ewing sarcoma	UA	7
Vasquez et al.	2017	Peru	78	Mixed	2; 150	NLR, PLR	OS	Osteosarcoma; Ewing sarcoma	UA; MA	6
Li et al.	2017	China	216	Mixed	2.65; 118	NLR, PLR	OS	Osteosarcoma	UA	7
Xia et al.	2016	China	359	Mixed	3.43; 122	NLR, PLR	OS, PFS	Osteosarcoma	MA	7
Liu et al.	2016	China	162	Mixed	2.57; 123.5 4.73	NLR, PLR LMR	OS	Osteosarcoma	MA	7
Ninna et al.	2016	Denmark	172	Nonmetastatic	5.3	NLR	OS	Osteosarcoma Ewing sarcoma Chondrosarcoma	MA	8
Liu et al.	2015	China	327	Mixed	3.43	LMR	OS, EFS	Osteosarcoma	MA	8

Abbreviations: NLR: neutrophil lymphocyte ratio; PLR: platelet lymphocyte ratio; LMR: lymphocyte monocyte ratio; OS: overall survival; PFS: progression-free survival; DFS: disease-free survival; EFS: event-free survival, MA: multivariate analysis; UA: univariate analysis.

**Table 2:** Subgroup analysis of the prognostic value of NLR for OS.

Subgroup analysis	No. of studies	*I* ^2^ (%)	HR	95% CI	*P*
OS Total	9	78.2	1.76	1.29-2.41	<0.001
Histology type					
Osteosarcoma	6^∗^	85.4	1.78	1.18-2.69	0.006
Ewing sarcoma	2	0	2.11	1.26-3.54	0.005
Chondrosarcoma	1	-	1.17	0.69-2.01	0.557
Mixed	1	-	2.2	0.96-5.02	0.061
Ethnicity					
Asian	7	83.1	1.66	1.17-2.36	0.005
Non-Asian	2	0	2.25	1.34-3.77	0.002
Analysis method					
MA	7^+^	81	1.7	1.16-2.49	0.006
UA	3	0	1.76	1.29-2.41	<0.001
Metastasis status					
With metastatic cases	8	79.6	1.73	1.25-2.4	0.001
Without metastatic cases	1	-	2.2	0.96-5.02	0.061

^∗^Vasquez et al. study reported Ewing sarcoma cohort and osteosarcoma cohort separately. +: Vasquez et al. study reported NLR of Ewing sarcoma cohort by means of MA and osteosarcoma cohort with UA. Abbreviations: NLR: neutrophil lymphocyte ratio; HR: hazard ratio; CI: confidence interval; MA: multivariate analysis; UA: univariate analysis.

**Table 3:** Subgroup analysis of the prognostic value of PLR for OS.

Subgroup analysis	No. of studies	*I* ^2^ (%)	HR	95% CI	*P* value
OS Total	7	69.6	1.32	0.99-1.75	0.059
Histology type					
Osteosarcoma	5	68.4	1.19	0.9-1.57	0.229
Ewing sarcoma	2	0	2.03	1.2-3.43	0.009
Ethnicity					
Asian	5	71.6	1.21	0.91-1.60	0.19
Non-Asian	2	0	2.27	1.15-4.49	0.019
Analysis method					
MA	4	56	1.08	0.83-1.39	0.569
UA	3	0	1.87	1.33-2.62	<0.001

Abbreviations: PLR: platelet lymphocyte ratio; HR: hazard ratio; CI: confidence interval; MA: multivariate analysis; UA: univariate analysis.

## Data Availability

The data used in this paper are available from the corresponding author upon reasonable request.
